# Adolescent Pregnancy Outcomes and Risk Factors

**DOI:** 10.3390/ijerph20054113

**Published:** 2023-02-25

**Authors:** Jana Diabelková, Kvetoslava Rimárová, Erik Dorko, Peter Urdzík, Andrea Houžvičková, Ľubica Argalášová

**Affiliations:** 1 Department of Public Health and Hygiene, Medical Faculty, University of Pavol Jozef Šafárik, Šrobárova 2, 041 80 Košice, Slovakia; 2 Department of Gynaecology and Obstetrics, Medical Faculty, Louis Pasteur University Hospital, University of Pavol Jozef Šafárik, Trieda SNP 1, 040 11 Košice, Slovakia; 3 Institute of Hygiene, Faculty of Medicine, Comenius University in Bratislava, Špitálska 24, 813 72 Bratislava, Slovakia

**Keywords:** teenage pregnancy, adolescent pregnancy, birth weight, preterm birth, neonatal outcomes, risk factors

## Abstract

One of the major social and public health problems in the world is adolescent pregnancy. Adolescent pregnancy is strongly associated to less favorable results for both the mother and the newborn. We conducted this research to ascertain the impact of teenage age on neonatal outcomes and also observed the lifestyles of pregnant teenage girls. We conducted a study of 2434 mothers aged ≤19 years (n = 294) or 20–34 years (n = 2140) who gave birth in 2019–2020 at the Department of Gynaecology and Obstetrics of Louis Pasteur University Hospital in Košice. The data on mothers and newborn infants have been reported from the reports on mothers at childbirth. Women between the ages of 20 and 34 served as the reference group. The teenage mothers were more likely to become pregnant if they were unmarried (OR = 14.2; 95% CI = 9.3–21.6; *p* < 0.001) and had a basic education or lack of education (OR = 16.8; 95% CI = 11.5–24.6; *p* < 0.001). Additionally, they were more likely to smoke when pregnant (OR = 5.0; 95% CI = 3.8–6.6; *p* < 0.001). Low birth weight was more common in newborns born to adolescent mothers than in those born to adult mothers (*p* < 0.001). Our findings showed that infants of teenage mothers often had lower birth weights (−332.6 g, *p* < 0.001). Adolescent mothers were associated with lower Apgar scores at the first minute (*p* = 0.003). As compared with the control group, pregnant teenage girls had a greater prevalence of preterm deliveries in our research (*p* = 0.004). This study finds significant age-related disparities in neonatal outcomes between mothers. These results might be used to identify vulnerable groups who need special assistance and actions to reduce the probability of negative outcomes for such groups.

## 1. Introduction

Adolescent pregnancies are a global public health problem. Teenage pregnancy is the pregnancy of 10- to 19-year-old girls [1]. Adolescents are further divided into early (10–14 years old), middle (15–17 years old), and late adolescents (over 17 years old) [2].

According to the World Health Organization, adolescent pregnancies are a global problem for both developed and developing countries. Although the global teenage birth rate has decreased, there are regional differences in the rates of change. Adolescent pregnancies have decreased globally, from 64.5 per 1000 women in 2000 to 42.5 per 1000 women in 2021. However, there are huge differences in levels between and within countries. While the estimated global teenage birth rate has decreased, the actual number of childbirths to teenagers continues to be high. Pregnancy in girls under the age of 19 is severe in every aspect and requires very complex and long-term solutions [1].

The transition from childhood to adulthood occurs during the phase of adolescence, during which there are numerous changes in the physiological, anatomical, structural, and psychological aspects. Because many teenagers are not physically or mentally prepared for pregnancy and childbirth, they are more likely to experience complications that can have serious health consequences. Giving birth during adolescence has serious consequences for the health of the mother and her infant [1]. The adolescent age group is associated with adverse pregnancy outcomes [2,3].

The rate of teenage pregnancies has recently been greatly affected by several significant factors. The decreasing age at menarche is one of the factors that can affect a woman’s fertility. Since the 19th century, the age at menarche has been decreasing at a rate of 2–3 months per decade in many European countries, resulting in an overall decrease of about 3 years. Most of the decrease in menarche age is related to better nutrition and health. The onset of first sexual activity occurs at a significantly younger age, which is another contributing element. Teenage pregnancy, therefore, remains a serious social, economic, and health problem [4].

Young maternal age is more likely a marker for one or more other maternal risk factors associated with poor birth outcomes. Poverty, low education, and inadequate family support are also problems. These factors increase the risk of sexually transmitted infections, unsafe abortions, and birth complications, all of which are exacerbated by inadequate prenatal care [5]. For girls, early pregnancies can have social consequences such as lower status in the household and community; stigmatization; abuse by family, peers, and partners; and early and forced marriage. Early pregnancy and childbirth during adolescence can hinder a girl’s otherwise healthy development into adulthood and negatively affect her educational opportunities, financial security, and health. Many teenage girls who are pregnant cannot continue their education or work because they are pregnant. This can have a big impact on their future [6]. In addition, children born to parents who cannot care for them face additional dangers. In the first few years of a child’s life, the mother–child relationship declines. This is primarily due to the mother’s immaturity. When teenage mothers are victims of sexual assault, the situation is even worse. Apart from their mothers, these children tend to be brought up by their grandparents and relatives, with frequent changes in caregivers. Children have a higher risk of being abused or neglected and a higher risk of failing in school and are more likely to engage in criminal behavior later on [7].

The economic, social, and political development and progress of any country depend on the healthy size of adolescents and children. As a result, the healthier the teenager is, the healthier the nation and future generations will be. Teenagers thus need special attention from us.

Understanding the issue is necessary to develop and carry out prevention initiatives to decrease teen pregnancy. Knowledge about the target groups, teenage pregnancy and birth outcomes, and the risk and preventive factors related to teenage pregnancy is needed. This information is important in choosing which risk and protective factors to target and, thus, better implementing the effective implementation of evidence-based adolescent pregnancy prevention practices.

Examining the newborn outcomes and risk variables associated with adolescent pregnancies was the aim of the present research.

## 2. Materials and Methods

The research took place in the years 2019–2020 in eastern Slovakia. This study included 2434 newborns and their mothers. Data were collected at the University of Pavel Jozef Šafárik’s Faculty of Medicine and the Louis Pasteur University Hospital’s Gynecology and Obstetrics Clinic in Košice. This hospital has a higher prevalence of mothers with high-risk pregnancies because it is the East Slovakian center for low birth weight and preterm birth.

The data were obtained from hospital records. Available information included the mother’s education, marital status, lifestyle, and when prenatal care began. Additionally, the Apgar scores at 1 and 5 min, the newborn’s gestational age, and the newborn’s weight were recorded. The total number of mothers in the results tables was different because not all the data for each mother were available in the clinical records. The study excluded women who were carriers of multiple pregnancies because they had a higher risk of preterm birth and lower birth weights of their newborns. Thus, women with multiple pregnancies were not included among the participants.

Maternal age was defined as the mother’s age in completed years at the time of delivery. The youngest women recruited to the cohort were 14 years old; therefore, the data for this study were limited to women aged 14–34 years at delivery who had a singleton pregnancy. The results for women under the age of 19 were compared with the results for women in the reference group (20–34 years). The age range of 20 to 34 years was chosen as the reference group because this age range had the lowest risk of developing age-related problems.

In our records, a woman who smoked at least one cigarette per day while pregnant was considered a smoker. All women who consumed 15 g of alcohol per day were considered alcohol consumers. This is equivalent to 0.5 L of 12-degree beer, 0.3 L of wine, or 0.5 dL of strong alcohol.

The neonatal outcome variables of interest in this study were low birth weight (less than 2500 g), very low birth weight (less than 1500 g), extremely low birth weight (less than 1000 g), macrosomia (birth weight greater than 4000 g), preterm birth (less than 37 weeks gestation), very preterm birth (less than 32 weeks gestation), extremely preterm birth (less than 28 weeks gestation), and low Apgar score at the first and fifth minutes (less than 7).

Most mothers completed eight prenatal care visits. Thus, we divided the group of mothers into two groups: those who had fewer than eight antenatal visits and those who had eight or more visits.

The IBM SPSS Statistics 23.0 program (IBM SPSS Statistics for Windows, Version 23.0. IBM Corp., Armonk, NY, USA) was used to analyze the data. The data were given as median (min–max), mean (standard deviation), and number (percent).

The data were processed using both primary characters and modified characters (categorized). Most of the findings were statistically significant, and the analysis included important discoveries that were related to the collected empirical data. The χ2 independence test, with a significance level of 0.05, was used to assess the frequency of individual variations of characteristics in the analyzed groups and subgroups. The Student’s *t*-test was used to compare the arithmetic means of continuous variables. The odds ratio, or, was used to compare the frequency of social and anamnestic variables in the adolescent mothers and mothers from the reference group.

## 3. Results

Data were available for 2434 pregnancies for this analysis. A total of 294 (12.1%) of these births included teenagers between the ages of 14 and 19. The controls were 27.9 ± 3.9 years old on average, whereas the adolescents’ mean age was 17.4 ± 1.4. Table 1 displays the characteristics of the study’s participants.

Our study demonstrated that adolescent mothers had lower levels of education (*p* < 0.001), only primary school (84.1%). About 46% of teenage girls reported smoking during pregnancy. In the reference group, the proportion of smokers was 14.6% (*p* < 0.001). Alcohol consumption during pregnancy was relatively low at 0.6%, and the data on alcohol use were not statistically significant (Table 1).

In the adolescent group, there were up to 45.7% of women (*p* < 0.001) who went to the doctor after the first trimester. Most mothers completed eight prenatal care visits. Therefore, we divided the group of mothers into two groups: those who had fewer than eight antenatal visits and those who had eight or more visits. Up to 75.9% of teenage girls who were pregnant had fewer than eight clinic visits (*p* < 0.001) (Table 2).

Adolescent girls were significantly more likely to be single (OR = 14.2; 95% CI = 9.3–21.6; *p* < 0.001), to have less education (OR = 16.8; 95% CI = 11.5–24.6; *p* < 0.001), and to smoke during pregnancy (OR = 5.0; 95% CI = 3.8–6.6; *p* < 0.01). They were more likely to visit a doctor for the first time during pregnancy after the first trimester (OR = 0.3; 95% CI = 0.2–0.3; *p* < 0.001) and were more likely to visit a doctor fewer than eight times (OR = 4.0; 95% CI = 3.0–5.3; *p* < 0.001) during pregnancy (Table 3).

Table 4 shows the results for newborns. Infants born to teenage mothers had a significantly higher rate of low birth weight than those born to women who were adults (*p* < 0.001). Our findings showed that children born to teenage mothers weighed less on average (−332.6 g, *p* < 0.001). In contrast to the control group, pregnant adolescents in our analysis had a higher prevalence of premature births (*p* = 0.004). Children of adolescent mothers had a lower first-minute Apgar score (*p* = 0.003).

## 4. Discussion

Pregnancy in adolescence is a health problem worldwide. Teenagers themselves are a high-risk group in need of high-priority interventions. In general, most pregnancies in adolescence are extra-marital and unintended [2]. The teenage mothers in this research were more likely to be single (OR = 14.2; 95% CI = 9.3–21.6; *p* < 0.001), which is similar to previous studies [5,8,9,10].

Psychological immaturity is common among adolescent mothers. Because they do not understand the value of family planning, they often engage in risky sexual behavior and become pregnant while still in school and still living with their parents [9]. This study confirms that teenage mothers are significantly more likely to have a low level of education (*p* < 0.001). These findings agreed with those of other research investigations carried out in other nations [2,9]. Adolescent girls often drop out of school due to pregnancy or childbirth. Sometimes problems at school and poor school performance appear even before pregnancy. Some teenage girls who are not doing well in school may find motherhood an attractive option. When these variables combine, young mothers have fewer career possibilities, often resulting in lower earnings for the rest of their lives [9,11]. Early pregnancies are significantly reduced by education; the more years of education, the lower the rate of early pregnancies [1].

Quitting smoking has a direct impact on the health of the fetus. Teenagers in our research were more likely to smoke during pregnancy (OR = 5.0; 95% CI = 3.8–6.6; *p* < 0.001). Previous research has shown that several high-risk activities are associated with a higher likelihood of pregnancy. These activities included the use of tobacco products, drinking alcohol, drug use, and risky sexual behavior [9,10,12,13,14].

Teenagers need accurate information about where to go when they need advice and help. Numerous studies have highlighted the benefits of prenatal care in minimizing pregnancy risks [9,15,16,17]. Unlike controls, pregnant adolescent girls in our study used prenatal care services less frequently. This was confirmed by a later gestational age at the first visit (*p* < 0.001) and a lower number of visits to the doctor during pregnancy (*p* < 0.001). This may be a result of a lack of information about the community services offered and the benefits of providing early and routine care. Teenagers may think they are not entitled to prenatal care, or they may choose to keep the pregnancy a secret [9,11,18]. Pregnant teenagers often interrupt school attendance, partly because of their participation in prenatal care. If clinic times are compatible with school attendance and medical staff are sensitive to adolescent needs, antenatal visits are more likely to be attended. Therefore, the needs of adolescents must be taken into account when providing prenatal care. However, direct study comparisons are difficult as there are different definitions of appropriate prenatal care. Regardless of how prenatal care is defined, the data suggest that adolescents tend to receive less adequate care than adult women [3,8,14,19,20,21]. Similar findings were obtained by Kassa et al. [22], who found that the number of antenatal care visits was lower in the teenage group and that doctor visits started later in pregnancy in this group. De Vienne et al. [23], on the other hand, did not find a difference between younger and older women in the analyzed age categories. Quinlivan and Evans published a study [24] comparing the outcomes of adolescents attending either a general or a specialist antenatal clinic for teenagers. In adolescent pregnancy clinics, prenatal care was provided by a multidisciplinary team and included social support and thorough infection screening. The rate of preterm births has decreased significantly as a result of the care provided at teen pregnancy clinics. According to the authors, the key strategies were the prevention of ascending infections of the genital tract and the provision of comprehensive treatment for teenagers. Healthcare professionals should be aware that teenage pregnancies are high-risk pregnancies and educate young women about the value of prenatal care and frequent antenatal visits.

In our study, preterm births were more common among pregnant teenage mothers than in controls (*p* = 0.004), which is similar to previous studies [25,26,27,28,29,30]. Due to the fact that preterm birth is a complex pregnancy problem, it is complicated to identify the exact cause. According to Debiec et al. [17], preterm birth is more common in teenagers who receive insufficient prenatal care, which supports the hypothesis that poor prenatal care is a risk factor for preterm birth. However, Chen et al. [4] point out that the risk of preterm birth persisted even in women who received adequate prenatal care. Yadav et al. [10] found that preterm birth was significantly more common in teenagers. According to them, the rise might be attributed to biological immaturity and socioeconomic deprivation. Clinically indicated preterm births may be the result of medical conditions such as intrauterine growth restriction or spontaneous labor. Both spontaneous preterm birth and intrauterine growth restriction are associated with maternal malnutrition, and there is strong evidence linking both conditions to maternal smoking during pregnancy [13,18,31,32,33,34,35]. Adolescent mothers are more likely to deliver preterm due to gynecological immaturity (such as a short cervix [25 mm] and a small uterine volume) and susceptibility to subclinical infections. Other studies suggest that these risks are related to biological immaturity in adolescent females and are not related to social deprivation, smoking, or inadequate prenatal care [27,36,37].

In this study, the percentage of low birth weight in infants born from adolescent mothers was higher than in mothers who gave birth in adulthood (*p* < 0.001), which is similar to previous studies [23,27,28,29,38,39]. It is thought that growing adolescents may compete with the fetus for resources, which might hinder fetal development and lead to low-birth-weight newborns or newborns that are small for their gestational age [40]. Marvin-Dowle et al. [40] conducted research in England among women aged 19 years and 20–34 years to examine the relationship between maternal and newborn outcomes in teenage women. Extremely low birth weight was found to be significantly more common in the teenager group compared with the control group.

Extremely underweight newborns have a higher risk of death within the first few months of life [12] as well as long-term problems with their physical and cognitive development [9,11]. Extremely low birth weight was not more common in our study cohort of adolescent mothers (*p* = 0.246).

The term Apgar, or appearance, pulse, grimace, activity, and respiration, was created by Doctor Virginia Apgar. This score is a simple method for evaluating neonates one and five minutes after birth. A newborn’s Apgar score is determined by several variables, including color, heart rate, reflexes, muscle tone, and breathing. Scores for each item range from 0 (zero), 1, or 2, with a total score of 7 to 10 considered good [41]. No significant difference in the low Apgar score between adolescent and adult pregnancies was found when compared with hospital-based retrospective cohort research in Nepal by Yadav et al. [10]. Due to several sociodemographic, obstetric, and dietary factors, low Apgar scores occur more frequently in teenage pregnancies than in adult pregnancies [3,22]. In a study conducted over 6 years in Japan with 30,831 women under the age of 25 who were pregnant with a singleton, Ogawa et al. [29] examined the relationship between adolescent pregnancy and adverse outcomes. They found that low Apgar scores were significantly more common among adolescent mothers than among mothers aged 20 to 24 [29]. Low Apgar scores are associated with infant complications such as breathing difficulties, feeding problems, hypothermia, and seizures [42]. Low Apgar scores at five minutes correlate with mortality and may indicate a higher likelihood of cerebral palsy [41]. In our study, the difference in the prevalence of low Apgar scores between adolescent mothers and the control group was confirmed only when the Apgar score was evaluated after the first minute (*p* = 0.003).

The development of social policy can be improved by having a thorough understanding of all these socioeconomic factors that influence teen pregnancy.

The first and most important step in strategies to reduce adolescent pregnancies and associated poor neonatal outcomes should be to “prevent it”. Measures to reduce the prevalence of teenage pregnancy also include increasing the importance of education. Although there are many different techniques to prevent a young girl from becoming pregnant, sexual abstinence is the only one that is 100% successful. This approach is the only one that ensures zero pregnancy risk and safeguards the adolescent from contracting any STDs. It is important to make teenagers aware of the responsibility that comes with sexual activity. The more information teenagers receive about this topic, the higher the chance that they will behave cautiously.

Teenagers should be educated about the negative consequences of teenage pregnancy, especially by their parents and at school. Building adolescents’ knowledge, skills, resilience, and aspirations through relationships and education helps them delay sexual activity until they are ready; enjoy healthy, consensual relationships; and use family planning methods. Schools may play a role by encouraging students to make mature decisions about their sex and by disseminating the knowledge needed to prevent adolescent pregnancy.

Teenagers are more likely to have their first sexual experience later in life if they and their parents have open discussions about relationships and sexual health from a young age. When parents spend time discussing sex and family planning with their children, they can have a significant impact on their decisions. Some parents have trouble talking about this topic. The barriers to parental communication include embarrassment, concern that discussion may encourage early sexual activity, and uncertainty about how to properly answer questions. Parents and all practitioners who come into contact with young people therefore need guidance on how to talk to them.

## 5. Conclusions

In conclusion, pregnancies in adolescents should be considered high-risk pregnancies. It is necessary to emphasize the need for comprehensive prenatal care for pregnant adolescent children because insufficient prenatal care can be harmful to both the mother and her fetus. Promoting early and thorough prenatal care is a key strategy if adolescent pregnancy outcomes are to be improved. Addressing teen pregnancy also requires a major effort by families, service providers, schools, faith-based and community organizations, recreation centers, policymakers, and youth. Teenagers should be educated about the negative consequences of teenage pregnancy, especially by their parents and at school. Our results confirm the relatively high prevalence of pregnant adolescent girls who smoked. Education should therefore also focus more on the risks associated with the use of substances during pregnancy.

The most important elements in preventing unwanted teenage pregnancies are a functional and stable family, good relations between parents, and good relations between parents and children. Parents should be the main source of information about sex. Adolescent pregnancy is not only a medical problem but also a social and societal problem, so society also plays an important role in preventing unwanted pregnancies, spreading awareness among young people, and holding them accountable for their actions.

## 6. Limitation

The conclusions of this study must be interpreted in light of limitations in the dataset and study design. For example, this study cannot adequately control for such factors as infectious exposure and drug use, which may differ between the groups.

## Figures and Tables

**Table 1:** Characteristics of the sample by maternal age.

Variable	≤19 n (%)	20–34 n (%)	Total n (%)	*p*-Value
Marital status				
Single	200 (88.5)	582 (34.1)	782 (40.4)	**<0.001**
Married	26 (11.5)	1108 (64.8)	1134 (58.6)	
Divorced/widowed	0 (0.0)	19 (1.1)	19 (1.0)	
Education				
Primary	180 (84.1)	382 (23.4)	562 (30.4)	**<0.001**
High school	34 (15.9)	682 (41.8)	716 (38.7)	
University	0 (0.0)	569 (34.8)	570 (30.9)	
Smoking during pregnancy				
No	147 (53.8)	1666 (85.4)	1813 (81.5)	**<0.001**
Yes	126 (46.2)	285 (14.6)	411 (18.5)	
Alcohol use during pregnancy				
No	269 (98.5)	1914 (99.5)	2210 (99.4)	0.062
Yes	4 (1.5)	10 (0.5)	14 (0.6)	

Numbers in bold indicate statistically significant values.

**Table 2:** Antennal care received by the respondents.

Variable	≤19 n (%)	20–34 n (%)	Total n (%)	*p*-Value
The first visit of a gynecologist				
First trimester	153 (54.3)	1716 (81.7)	1869 (78.5)	**<0.001**
Later	129 (45.7)	384 (18.3)	513 (21.5)	
Visits to prenatal counseling				
≥8	68 (24.1)	1177 (56.0)	1245 (52.3)	**<0.001**
<8	214 (75.9)	923 (44.0)	1137 (47.7)	

Numbers in bold indicate statistically significant values.

**Table 3:** OR of various risk variables for adolescent mothers’ reproductive outcomes.

Variable	OR	95% CI	*p*-Value
Non-married vs. married	14.2	9.3–21.6	**<0.001**
Education basic vs. more	16.8	11.5−24.6	**<0.001**
Smoking during pregnancy yes/no	5.0	3.8−6.6	**<0.001**
Alcohol use during pregnancy yes/no	2.9	0.9–9.3	<0.062
The first visit of a gynaecologist later/first trimester	0.3	0.2−0.3	**<0.001**
Visits to prenatal counselling <8/more	4.0	3.0–5.3	**<0.001**

OR—odds ratio; CI—confidence interval. Numbers in bold indicate statistically significant values.

**Table 4:** Neonatal outcomes.

Variable	≤19 n (%)	20–34 n (%)	Total n (%)	*p*-Value
Preterm delivery (<37 weeks)	74 (25.2)	383 (17.9)	457 (18.8)	**0.004**
Very preterm delivery (<32 weeks)	13 (4.4)	77 (3.6)	90 (3.7)	0.508
Extremely preterm delivery (<28 weeks)	1 (0.3)	28 (1.3)	29 (1.2)	0.246
Low birth weight (<2500 g)	71 (24.1)	310 (14.5)	381 (15.7)	**<0.001**
Very low birth weight (<1500 g)	16 (5.4)	88 (4.1)	104 (4.3)	0.282
Extremely low birth weight (<1000 g)	2 (0.7)	32 (1.5)	34 (1.4)	0.423
Apgar score at 1 min <7	43 (14.6)	192 (9.0)	235 (9.7)	**0.003**
Apgar score at 5 min <7	17 (5.8)	90 (4.2)	107 (4.4)	0.224

Numbers in bold indicate statistically significant values.

## Data Availability

The data presented in this study are available on request from the corresponding author.
